# Parallelism and non-parallelism in diabetic nephropathy and diabetic retinopathy

**DOI:** 10.3389/fendo.2024.1336123

**Published:** 2024-02-14

**Authors:** Shanshan Tang, Xuedong An, Wenjie Sun, Yuehong Zhang, Cunqing Yang, Xiaomin Kang, Yuting Sun, Linlin Jiang, Xuefei Zhao, Qing Gao, Hangyu Ji, Fengmei Lian

**Affiliations:** ^1^ College of Traditional Chinese Medicine, Changchun University of Traditional Chinese Medicine, Changchun, China; ^2^ Guang’an Men Hospital of China Academy of Chinese Medical Sciences, Beijing, China; ^3^ Fangshan Hospital, Beijing University of Chinese Medicine, Beijing, China

**Keywords:** diabetic retinopathy, diabetic nephropathy, parallelism, non-parallelism, mechanism

## Abstract

Diabetic nephropathy (DN) and diabetic retinopathy (DR), as microvascular complications of diabetes mellitus, are currently the leading causes of end-stage renal disease (ESRD) and blindness, respectively, in the adult working population, and they are major public health problems with social and economic burdens. The parallelism between the two in the process of occurrence and development manifests in the high overlap of disease-causing risk factors and pathogenesis, high rates of comorbidity, mutually predictive effects, and partial concordance in the clinical use of medications. However, since the two organs, the eye and the kidney, have their unique internal environment and physiological processes, each with specific influencing molecules, and the target organs have non-parallelism due to different pathological changes and responses to various influencing factors, this article provides an overview of the parallelism and non-parallelism between DN and DR to further recognize the commonalities and differences between the two diseases and provide references for early diagnosis, clinical guidance on the use of medication, and the development of new drugs.

## Introduction

The International Diabetes Federation (IDF) Diabetes Map 2021 shows that the world’s adult diabetic population has reached 537 million in 2021, while the adult population with the disease grows by 1.7% to a projected 783 million in 2045 (1). Complications of diabetes mellitus can be categorized into acute and chronic complications, and chronic complications caused by long-term exposure to hyperglycemia can be categorized into microangiopathy, macrovascular disease, and neuropathy (2, 3). Among them, diabetic nephropathy (DN) and diabetic retinopathy (DR), as highly prevalent microvascular complications of diabetes, have become the leading causes of end-stage renal disease (ESRD) and blindness in adult patients, and the disease puts enormous economic pressure on the healthcare system as well as on the patients themselves (4–7). The study provides a comprehensive and up-to-date assessment of the current and 2045 global prevalence of DR through the largest meta-analysis to date, showing that the global prevalence of DR in the diabetic population is estimated to be 22.27%, in 2020, the number of adults with DR will be approximately 103.12 million globally. It will increase by 55.6% to 160.5 million in 2045 (6). DN is now the leading cause of ESRD in the adult working population, accounting for approximately 50% of developed countries (7). A meta-analysis of the prevalence of comorbid DN in the Chinese type 2 diabetes mellitus type 2 (T2DM) population (79,364 study subjects) showed that nearly 1/5 of diabetic patients have comorbid DN (8). It has been reported that type 1 diabetes mellitus (T1DM) patients with DR and T2DM patients without DR are 13.39 and 3.51 times more likely to develop DN, respectively. The prevalence of DN increases with the increasing severity of DR, and the two diseases are closely related (9). A cross-sectional study based on 26,809 patients in five primary hospitals in China showed prevalence rates of DN and DR of 32.3% and 34.6%, respectively, with a positive predictive value of 47.4% for DN (10). Increases in DR severity were strongly associated with increases in DN severity (11).

DN and DR have parallelism and non-parallelism in disease onset and progression. The parallelism of the two diseases includes the mutual predictive effect of predicting the development of the other diseases through one of them, the four aspects of common causative factors, similar pathogenesis, and common use of medication. Non-parallelism includes asynchrony of onset, variability of pathogenic factors, different pathogenic mechanisms, and medications. Exploring the parallelism of two diseases can, on the one hand, predict the development of the other diseases through one disease and take timely and effective preventive measures to prevent the development and deterioration of the disease; on the other hand, the use of generic drugs to treat the two diseases through the parallelism of the diseases can reduce the use of drugs, optimize the management of the disease and the use of medication, and alleviate the financial burden of the patients and the country. This paper reviews the parallelism and non-parallelism of the two diseases, recognizes the commonalities and differences between the two diseases, and guides the selection of clinical drugs, with a view to early diagnosis, delaying the onset and progression of the two diseases, improving patients’ quality of life, and reducing the burden on society.

## Parallelism between DN and DR development

Microvascular pathological changes in diabetes mellitus include the thickening of capillary basement membranes due to hyperglycemia (12), increased permeability and dysfunction of endothelial cells (13–15), and vascular smooth muscle cell dysfunction (16, 17). Despite their functional differences, the kidney and the eye share similarities in developmental pathways and molecular structure (18, 19); the glomerulus and choroid have similar structures and vascular networks (19); the internal blood–retina barrier (BRB) and the glomerular filtration barrier have similar developmental pathways (20); the renin–angiotensin–aldosterone system (RAAS) found in both organs, the eye and the kidney (21, 22), leads to parallelism in their pathogenesis. In the following, the parallelism of the two diseases’ development will be described in terms of their mutual predictive effects, disease-influencing factors, identical pathogenesis, and common medication.

### Reciprocal predictive effects of DN and DR

DN and DR are closely related as both microvascular complications of diabetes mellitus (23). The severity of renal impairment in DN correlates with the severity of ocular damage in DR (24). The presence of DR can be diagnosed by fundus examination. In contrast, the kidneys are hidden in the abdomen of the human body, so doctors cannot accurately see the specific situation, and the gold standard for diagnosing renal disease is renal puncture biopsy (25). However, renal puncture biopsy is an invasive test, which is often difficult for the majority of patients to accept, so the use of fundus examination to predict renal disease is increasingly becoming the primary means of clinical doctors to screen for DN. At the same time, proteinuria in patients with DN is also often used to predict the severity of DN and predict the development of DR.

#### DN predicts DR

In a cross-sectional study, 250 T2DM patients with DN diagnosed by renal biopsy were divided into two groups: 130 patients in the DN without DR group and 120 patients in the DN and concomitant DR group. Logistic regression analysis was performed on the above 250 patients to clarify the risk factors for DR, and the results showed that the risk of DR was significantly associated with the risk of developing DR by proteinuria, hematuria, estimated glomerular filtration rate (eGFR) baseline correction, glomerulopathy severity, and DM history >10 years were significantly associated with the risk of developing DR (26). Through a cross-sectional study of 1,102 T2DM patients aged not less than 30 years recruited in Korea in 2010–2011, the results showed that grouped according to DR severity, non-proliferative diabetic retinopathy (NPDR), severe NPDR, and proliferative diabetic retinopathy (PDR), with their early morning field urine samples retained for urinary albumin–creatinine ratio (ACR) measurement, the results showed that the optimal cutoff value of ACR for predicting DR was 2.26 mg/mmol (20 μg/mg), and as the severity of DR increased, ACR ≥ 2.26 mg/mmol tended to increase, and the risk of severe NPDR and PDR also increased, demonstrating that ACR is an independent risk factor for DR (27). Also, in DR patients, even in the absence of proteinuria, we can predict subclinical DN based on eGFR (11).

#### DR predicts DN

DR was found to be associated with an increased risk of DN prevalence in patients with T2DM through a prospective study. However, the predictive value of DR for the risk of DN in patients with T2DM was relatively low, possibly since the predictive value of DR for DN may be affected by aspects such as mean age, proportion of men, and study quality (28). In a 25-year follow-up based on a cohort study of 184 patients from Denmark, it was suggested that PDR in patients with T1DM may be an independent marker of long-term nephropathy development (29). A deep learning model validated with 115,344 retinal fundus photographs from 57,672 patients indicates that chronic kidney disease staging and eGFR prediction can be performed by retinal fundus image testing and that the model evaluates the level of agreement between the algorithm’s predicted GFR and the measured eGFR by means of a Bland–Altman plot. The results showed that the artificial intelligence (AI) model was able to extract the information for predicting GFR and embed it skillfully in the fundus images, and the deployment of AI fundus diagnostic system can be used as a non-invasive, high-throughput, and low-cost screening tool in the early stage of renal disease regardless of geographic display to effectively predict the progression of renal disease (30). A cross-sectional study shows that DR, especially PDR, is an independent predictor of kidney disease (9).

### Risk factors associated with DN and DR

Age, male gender, hypertension, diabetes duration, diabetic neuropathy, DN, diabetic foot ulcers, and foot amputation were used as independent risk factors for DR based on two cross-sectional studies in urban hospitals in China (31, 32). A cross-sectional study based on 13,473 diabetic patients complemented postprandial glucose, glycosylated hemoglobin (HbAlc), triglycerides, and low-density lipoprotein as independent risk factors for DR, with preprandial glucose, postprandial glucose, and HbAlc all being significant predictors of the development or progression of DR (33), which showed that each 1% increase in HbAlc was associated with a 40% increase in the risk of microvascular events (34). In addition, anemia (35, 36), pregnancy (37), smoking (38), genetic factors (39), and microalbuminuria (40, 41) have been shown in multiple trials to increase the risk of DR. Meta-analysis of multiple prospective cohort studies suggests that obesity is a risk factor for DR (42, 43).

Studies have shown that elevated blood glucose levels, long duration of diabetes, hypertension, obesity, and dyslipidemia can contribute to the development of DN (44). Elevated microalbuminuria levels increase the risk of DN (45, 46). Numerous epidemiologic investigations have shown that genetic susceptibility contributes to the development of DN (47–49). A systematic evaluation and meta-analysis based on 20 cohorts identified age, body mass index, smoking, DR, glycosylated hemoglobin, systolic blood pressure, high-density lipoprotein, triglycerides, and urinary albumin/creatinine ratio as risk factors for the development of DN (50). The study results show a substantial overlap in the causative risk factors of the two diseases.

### Similar pathogenesis of DN and DR

**Figure 1:**
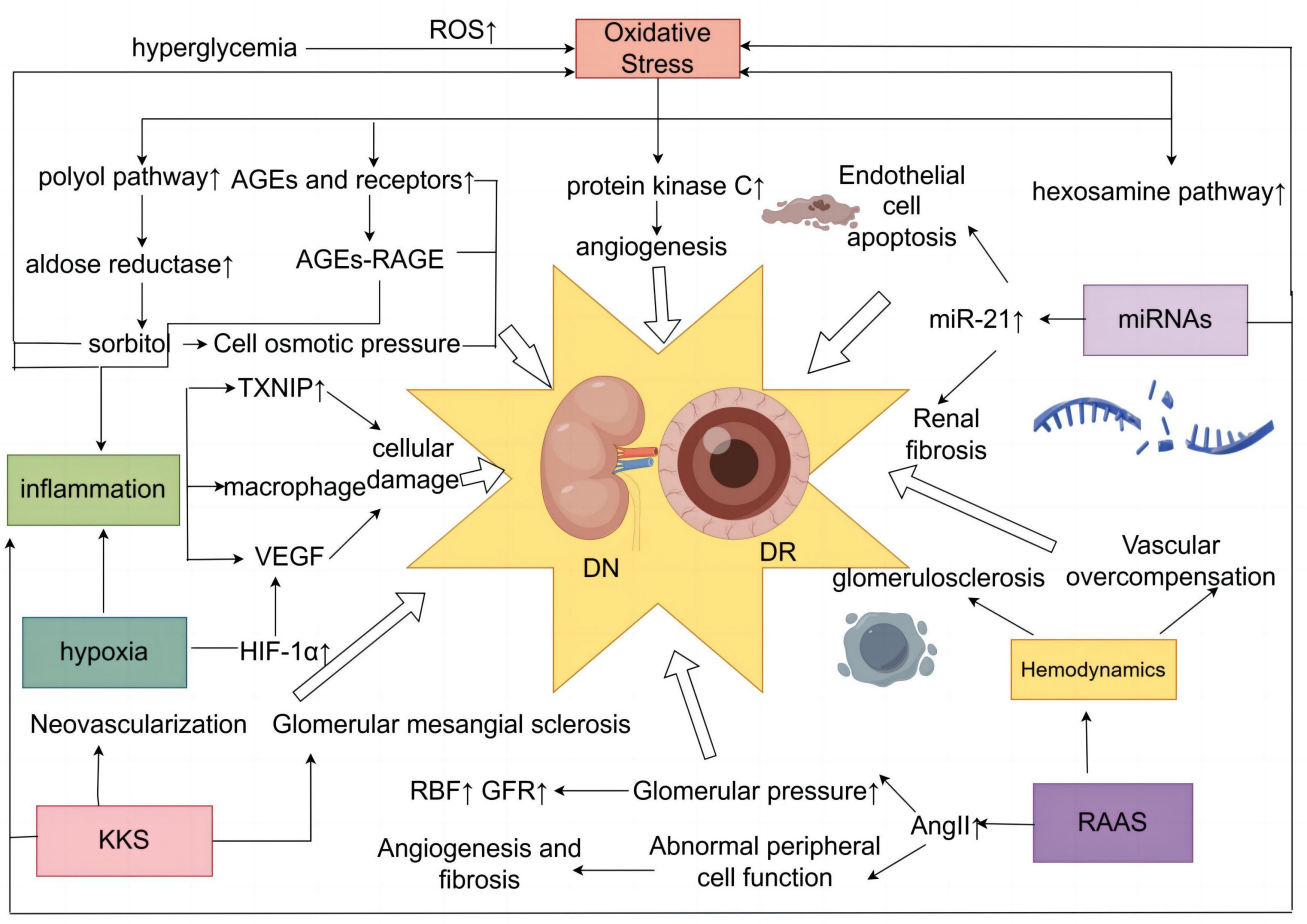
Diagram of the common mechanism of DN and DR. DN, diabetic nephropathy; DR, diabetic retinopathy.

DN and DR are microvascular complications of diabetes mellitus, and the pathological features of retinal tissue in patients with DR include loss of pericytes and endothelial cells, destruction of BRB, and retinal neovascularization (51, 52). Pathological changes in DN may include glomerular tunica dilatation, basement membrane thickening, glomerular endothelial injury, podocyte injury, and tubular junction abnormalities (53–56). Diabetes-induced endothelial dysfunction in microangiopathy is characterized by decreased nitric oxide (NO) bioavailability, increased oxidative stress, imbalance between vascular endothelial growth factor (VEGF) and NO, and impaired endothelial function repair (57). In the diabetic microvascular system, intracellular hyperglycemia induces vascular endothelial injury through various pathophysiological processes, including inflammation, endothelial cell–reticulocyte/pericyte conversation, and exocytosis (58). In addition, several key regulators such as cell adhesion molecule (CAM), VEGF family, and Notch signaling are involved in developing DN and DR diseases (58). Since the eye and the kidney share similarities in organizational structure and physiological function, exposure to the same risk factors often leads to the occurrence of the two diseases, with simultaneous similarities in pathogenesis (**Figure 1**).

#### Oxidative stress

Oxidative stress plays a vital role in the development of microvascular and cardiovascular complications in diabetes mellitus (59). Excessive production of reactive oxygen species (ROS) is a major cause of oxidative stress (60–62). Hyperglycemia plays a central role in the development of microvascular complications in diabetes mellitus (63), with a 37% reduction in the incidence of microvascular end-point events for every 1% reduction in HbAlc (64), and a sustained state of hyperglycemia in the cell induces an overproduction of mitochondrial ROS (65). This increased production of ROS is a central and primary mediator of diabetic tissue damage, with this single upstream mediator leading to the activation of five major pathways involved in the pathogenesis of complications, including activation of the polyol pathway (66), increased formation of advanced glycosylation end products (AGEs) (67), increased expression of receptors for AGEs and their activating ligands (68), activation of protein kinase C (PKC) isoforms (69), and enhancement of the hexosamine pathway (70), which are present in the mechanisms of DN and DR production (65, 71) Hyperglycemia-induced ROS triggers renal fibrosis and inflammation and causes significant tissue damage by promoting lipid peroxidation, DNA damage and protein modification, and mitochondrial dysfunction, inducing DN (72). Overproduction of ROS in the diabetic retina leads to retinal cell damage by altering cell signaling, ultimately leading to DR (73).

AGEs aggregated in the hyperglycemic state play an important role in DR (74). AGEs can induce the cross-linking of extracellular matrix proteins through direct action, leading to endothelial dysfunction and neural and vascular components (75, 76). AGEs also interact with specific receptors and binding proteins to induce oxidative stress and cellular dysfunction involved in the progression of DN (77). The polyol pathway contains aldose reductase (AR), mainly found in tissues such as the retina, lens, and kidney (78). Abnormally elevated blood glucose can lead to activation of the AR, which oxidizes glucose to sorbitol and can induce or exacerbate intracellular oxidative stress (79–81), promoting diabetic microangiopathy. Hyperglycemia triggers the glycolytic pathway, which further enhances the synthesis of di-glycerol (DAG), and PKC is activated as a target protein kinase for di-glycerol (82). Total DAG levels in vascular tissues are increased in the retina (83, 84) and glomeruli (85, 86) of diabetic patients. PKC activation impairs glomerular blood flow and filtration, causing renal hypertrophy and glomerular hyperplasia and contributing to the onset and progression of DN, which is mainly characterized by proteinuria (87, 88). PKC-mediated upregulation of extracellularly regulated protein kinases ERK1/2 and matrix metalloproteinases (MMPs) accelerates cell proliferation, migration, maturation, and formation of new blood vessels (89). PKC activation affects blood flow, causes retinal microvascular constriction, induces neovascularization, and promotes the development of DR (90).

#### Inflammatory response

Chronic subclinical inflammation underlies the vascular pathology of DR, and the stimulated inflammation of leukocyte adhesion to the retinal vascular system and the role of leukocytes in leukocyte stasis with risk of capillary closure suggest that this condition may represent a form of low-grade inflammation (91, 92). A study has shown that DR vascular leakage and no perfusion are temporally and spatially associated with retinal leukocyte stasis in a streptozotocin-induced diabetic rat model (93). NLRP1, NLRP3, NLRC4, and AIM2 inflammatory vesicles can lead to DR pathogenesis (94). DN is a chronic low-grade inflammatory disease (95, 96). Proinflammatory cytokines, chemokines and their receptors, adhesion molecules, and transcription factors are involved in the development and progression of DN (97). Low-grade inflammation accumulates extracellular matrix in the glomerular basement membrane by stimulating endothelial cells and podocytes, while excessive extracellular matrix deposition can cause an acute phase reaction (98). Nuclear factor κB (NF-κB), one of the major factors involved in the inflammatory response, is a crucial link in regulating chemokines, cell adhesion proteins, inflammatory cytokines, and other molecules associated with DN mechanisms (99). Meanwhile, thioredoxin-interacting protein (TXNIP), macrophages, and various cytokines are involved in the pathogenesis of DN and DR.

TXNIP is highly expressed in diabetic retina (100–103). TXNIP, as an early response gene in the high-glucose environment, can induce AGE receptor expression; at the same time, high glucose-induced TXNIP expression enhances AGE receptor expression, leading to feedback in the TXNIP expression loop, which produces persistent chromatin remodeling and sustained inflammatory gene expression in diabetic retinal capillary endothelial cells (101), which can induce NLRP3 expression, increase IL-1β production in Müller cells (103, 104), and promote DR development. At the same time, hyperglycemia upregulates TXNIP expression and induces ROS production and inflammatory and fibrotic responses in diabetic kidneys, leading to dysregulation of autophagy and contributing to the development of DN (101, 105). Macrophages were recruited in rat glomeruli early after the onset of hyperglycemia (106). Macrophages cause tissue damage and sclerosis by producing ROS, cytokines, and proteases (107, 108). Macrophage-restricted protein tyrosine phosphatase 1B (PTP1B) is a crucial regulator of inflammation in the metabolic syndrome resulting from insulin resistance, and aberrant regulation of PTP1B may underlie retinal microvascular disease (109). Several cytokines are involved in the pathogenesis of DN and DR, among which VEGF is one of the essential cytokines (110, 111). Activation of VEGF leads to neovascularization and glomerular injury, and loss of late-stage podocytes induces a decrease in VEGF signaling, which can trigger vascular thinning and renal fibrosis and promote the DN process (58). The generation of neovascularization and enhanced vascular permeability in the retina are associated with the upregulation of VEGF, which is involved in pathological retinal neovascularization and increased vascular permeability, and its mechanism of action is to induce vascular endothelial cells to divide and proliferate through the action of secreting high levels of affinity receptors on retinal vascular endothelial cells (112, 113).

#### Hemodynamic changes

Early diabetes is characterized by increased blood flow, which is reflected in the kidneys as glomerular hyperfiltration (114). Abnormally elevated blood glucose leads to increased permeability of the glomerular filtration membrane, which results in an increased glomerular filtration rate (115). Hyperfiltration and hyperperfusion can stimulate the proliferation of glomerular mesangial cells, increase the mesangial matrix, damage the endothelial cells, and increase platelet aggregation, leading to the formation of micro-arteriolar flow. It can also cause inflammatory cell infiltration, increase the apoptosis of the mesangial cells, and so on. Thus, glomerulosclerosis is constantly progressing, and the loss of renal units is progressive, inducing the DN (22, 115). High blood glucose levels in the RAAS and its elevated angiotensin levels led to a slowing of renal blood flow, impaired glomerular filtration barrier, and proteinuria, leading to DN. At the same time, the pathological changes of DN can lead to renal small, micro-arteriolar sclerosis and micro-arteriolar constriction enhancement, so the renal blood flow is reduced, and glomerular sclerosis is exacerbated, leading to renal insufficiency (22, 116). Studies have shown that diabetic patients have elevated blood and serum viscosity and elevated red blood cell aggregation and adhesion, which increase the resistance to blood flow and reduce its stability in the flow process, leading to slow blood flow and decreased blood flow rate and triggering circulatory disorders (117). From a hemodynamic perspective, ocular blood flow is determined by the balance between ocular perfusion pressure and vascular resistance (118). Prolonged hyperglycemia leads to endothelial dysfunction, increased vascular resistance, abnormal retinal perfusion, and exacerbation of retinal ischemia and hypoxia (119). As feedback from local ischemia, excessive ischemic dilatation as well as uneven capillary resistance cause other vessels to undergo overcompensation, resulting in a vicious cycle that promotes the development of DR (120).

#### Hyperactivation of RAAS

Increased RAAS activity is an essential factor in the development of DN, and the end product of this system is angiotensin II (AngII), whose damaging effects include vasoconstriction, increased aldosterone secretion, growth, fibrosis, thrombosis, inflammation, and oxidative (121). Local expression of RAAS is upregulated by glomerular capillary hypertension associated with hyperglycemia-induced hyperfiltration state (122). Hyperactivation of RAAS leads to renal injury by pro-fibrotic and pro-inflammatory factors (123). High glucose leads to overactivation of the RAAS, causing AngII to activate AT1 receptors, and AngII is involved in glucose- and lipid-induced oxidative stress, inflammation, and apoptosis via AT1 receptors. It produces elevated glomerular hydrostatic pressure, proteinuria, and structural damage with sclerosis and fibrosis and promotes the development of DN (124, 125). Studies have shown that patients with DR have higher than average concentrations of reninogen, renin, and AngII in the vitreous humor (126). The presence of its own independent RAAS in the retina regulates blood flow and intraocular pressure (IOP) (127); its key effector molecule, AngII, modulates pericyte function (128); the retinal RAS is activated to stimulate growth factors, such as VEGF, which leads to vascular leakage, pericyte migration, angiogenesis, fibrosis, and exacerbation of DR (129).

#### Kallikrein–kinin system

The kallikrein–kinin system (KKS) exerts multiple effects on vascular and neuronal tissues through activation of B1 and B2 receptors, which are constitutively expressed in most tissues, and B1 receptors, which are induced to be expressed under stress conditions such as inflammation and diabetes mellitus (130, 131). In the eye, the KKS is one of the identified pathways in the vitreous of patients with PDR and diabetic macular edema (DME) (132). The KKS plays a key role in the pathophysiology of DR, including vascular inflammation and hyperpermeability, oxidative stress, vasodilation, retinal thickening, and neovascularization (133). Activated plasma kinin-releasing enzymes in the retinal and vitreous fluids of patients with PDR may directly contribute to the development of interstitial swelling and macular edema in these patients (134). In the kidney, B2 receptor deficiency has been shown to increase albumin excretion and glomerular thylakoid sclerosis in diabetic mice starting with the Akita mutation (130). Diabetic test mice lacking bradykinin B2 receptors show a significant increase in dominant albuminuria with increasing glomerular mesangial sclerosis (135). Bradykinin has been shown to have NO-releasing properties, induce endothelium-dependent vasodilation, exert antifibrotic and antihypertrophic effects, and stimulate glucose uptake (136). The study suggests a novel role for the KKS in preventing salt-induced and hypertension-induced kidney injury by inhibiting oxidative stress and inflammatory responses (137).

#### MiRNAs

MiRNAs play an essential role in the pathogenesis of microvascular diabetic complications DR and DN by regulating various pathways including inflammation, apoptosis, and oxidative stress (138). Growing evidence suggests that altered genomic DNA methylation, chromatin histone modifications, and non-coding RNA dysregulation are involved in the pathogenesis of DN (139). Dysregulation of miRNAs promotes DR progression by affecting pathways such as inflammation, oxidative stress, endothelial apoptosis, and vascular cell function (138).

Studies have shown that plasma miR-21 expression is elevated during the development of T2DM combined with DR and can be used to indicate the severity of T2DM combined with DR (140). The high glucose-induced upregulation of miR-21 expression in retinal endothelial cells indirectly regulates NF-κB expression and promotes apoptosis in retinal endothelial cells (141), as well as having a pro-angiogenic effect (142, 143). Serum and renal tissue miR-21 was found to be significantly elevated with the progression of DN (144). MiR-21 promotes epithelial–mesenchymal transition (EMT) and extracellular matrix (ECM) deposition through the downregulation of bone morphogenetic protein 7 (BMP-7), and EMT and ECM deposition in renal tubular epithelial cells is critical for DN pathogenesis (145). Significant histopathological changes in miR-21 expression in DN kidney biopsies can fully reflect the key role of miR-21 in the development of DN (146). Meanwhile, the pathogenic role of miR-21 in DN can also manifest as renal fibrosis (147) and inflammation (148).

#### Hypoxia

Hypoxia plays a vital role in diabetic complications and occurs in tissues central to the development of diabetes (pancreatic β-cells and adipose tissue) and in tissues susceptible to diabetic complications (nerves, retina, heart, blood vessels, kidneys, and wounds) (149). Hypoxia-inducible factor 1α (HIF-1α) gene polymorphisms may be associated with diabetes and diabetic complications (150). HIF-1α is a transcription factor that is expressed in response to decreased cellular oxygen partial pressure and activated to participate in angiogenesis, glycolysis, and regulation of vascular tone (151) The cellular adaptive response to hypoxia is mediated by hypoxia-inducible factor-1α, and hyperglycemia leads to decreased stability and low transcriptional potency of the HIF-1α protein in response to hypoxia (152), which also leads to a pseudo-hypoxic state that activates HIF-1α activity to adapt to hypoxia (153). Hypoxia is present in the diabetic kidney (154, 155), and renal tubular hypoxia is due to a combination of increased energy demand and decreased perfusion with non-hypoxia-related forces that drive the development of tubular atrophy and interstitial fibrosis, promoting the progression of DN in a vicious cycle (156). The maintenance of normal retinal function depends on a continuous supply of oxygen and the ability to detect and respond to localized hypoxia rapidly (157). Hypoxia occurs frequently during the development of DR (158, 159), and hyperglycemia-induced endothelial dysfunction, as well as inhibition of endothelial NO synthase and disturbances in vascular self-regulation, can lead to the development of hypoxia in retinal tissue (160). Oxygen (O_2_) is essential for the retina (161, 162), and reduced retinal blood flow in diabetic patients can lead to retinal hypoxia (163). Retinal hypoxia can cause neuroretinal dysfunction and degeneration, which directly lead to vision loss (164) and promote the development of DR.

### Co-medication of DN and DR

Because of the similarity between DN and DR in terms of pathogenic factors and pathogenesis, there is partial concordance in terms of the clinical use of drugs. Active control of blood glucose, blood pressure, blood lipids, and other related risk factors can slow down the development of the disease process, so the treatment of the two diseases used in the choice of glucose control drugs, antihypertensive drugs, lipid regulating drugs, and other related drugs have essential consistency (165). In addition, some of the drugs also have the effect of DN and DR at the same time, and the therapeutic effects of some of the drugs are listed below.

#### Inhibition of aberrant activation of the polyol pathway: the aldose reductase inhibitor epalrestat

AR plays a crucial role in the etiology of long-term diabetic microvascular complications such as retinopathy, nephropathy, and neuropathy (166). Epalrestat is a carboxylic acid derivative that inhibits aldose reductase in the polyol pathway, and the ability of epalrestat to reduce intracellular sorbitol accumulation has been linked to the pathogenesis of microvascular complications in diabetes mellitus (167). A 3-year multicenter aldose reductase inhibitor-diabetic complications trial in Japan, which included 594 patients, showed that epalrestat was particularly effective in patients with reasonable glycemic control and mild microvascular disease (168). Patients treated with epalrestat have also been shown to slow the progression of diabetic retinopathy–nephropathy, possibly due to the inhibition of oxidative and inflammatory stress through the inhibition of the polyol pathway by epalrestat (169).

#### Antioxidant: α-lipoic acid

α-Lipoic acid (ALA) is a potent antioxidant (170–172) and is valuable in preventing and treating ocular complications such as diabetic keratopathy and DR (170, 173, 174). ALA has insulin-mimetic and anti-inflammatory activities (175), which increase insulin sensitivity (176), prevent retinal lipid peroxidation in early diabetes (177), inhibit the activation of NF-κB induced by late glycosylation end products in endothelial cells (178), and inhibit apoptosis in retinal capillary cells (179), thereby controlling the development of DR. ALA attenuates oxidative stress by regulating the expression of enzymes involved in the formation of reactive oxygen species (180), prevents glomerular thylakoid matrix expansion (181), inhibits diabetic renal fibrosis by improving mitochondrial function and regulating the expression and activation of isotretinoin receptor α (RXRα) (182), and protects the kidneys by possibly inhibiting neutrophil infiltration, modulating inflammatory mediators (183), and delaying the development of DN.

#### Inhibitors of the RAS system: angiotensin-converting enzyme inhibitors and angiotensin II receptor blockers

Angiotensin-converting enzyme inhibitor (ACEI) versus angiotensin II receptor blocker (ARB) as first-line therapy for hypertension in DN patients presenting with proteinuria has been shown to have renal and cardiac-related benefits (184). Studies have shown that RAS induces various tissue responses, including vasoconstriction, inflammation, oxidative stress, cellular hypertrophy, proliferation, angiogenesis, and fibrosis, triggering diabetic microangiopathy (185). Mechanisms of ACEI and ARB analogs for DR are mostly related to the RAS system (186). RAS inhibition is the single most effective treatment for slowing the progression of diabetic nephropathy (187). Some experiments indicated that captopril, an ACEI representative drug, and chlorosartan, an ARB representative drug, inhibited hyperglycemia-induced leukocyte retention in the retinal vasculature in rats at 6 weeks and 1 week, respectively; that AngII-induced expression of vascular cell adhesion molecules was inhibited by chlorosartan in experimentally cultured human retinal endothelial cells; that captopril blocks capillary degeneration in the early stages of DR; and that the two drugs inhibit the development of DR by inhibiting the RAS system, which can block the early retinal capillary degeneration and the inflammatory response (188). Meanwhile, an animal study has shown that captopril can reduce oxidative stress in DR patients (189).

#### Vasoprotective drug: calcium 2,5-dihydroxybenzenesulfonate

Calcium 2,5-dihydroxybenzenesulfonate (CaD) is considered a vasoprotective drug that alleviates microcirculatory and hemorheological abnormalities (190), attenuates diabetes-induced endothelial dysfunction and inflammation (191, 192), and reduces diabetic vascular complications by interfering with acetyl heparin sulfate binding sites to decrease VEGF signaling (193). A systematic review and meta-analysis supports the validity of CaD’s multi-targeted effects on DR through antioxidant, anti-free radical, and vasoprotective effects and inhibition of inflammatory cytokines (194). CaD, as an antioxidant and microvascular protector, has significantly improved the renal impairment of DN. CaD controls the development of DN by alleviating vascular damage, improving microcirculation, delaying renal fibrosis, enhancing the barrier effect on glomerular capillary filtration, preventing or reducing the thickening of the glomerular capillary basement membrane, preventing the disruption of the podocyte process, and maintaining the integrity of the glomerular basement membrane, which then protects the glomerular filtration barrier of DN from being further disrupted and improves the glomerular terminal filtration function (195). CaD prevents DN by downregulating the key apoptotic factor Bim and inhibiting apoptosis in renal proximal tubular epithelial cells (196).

## Non-parallelism of DN and DR

**Figure 2:**
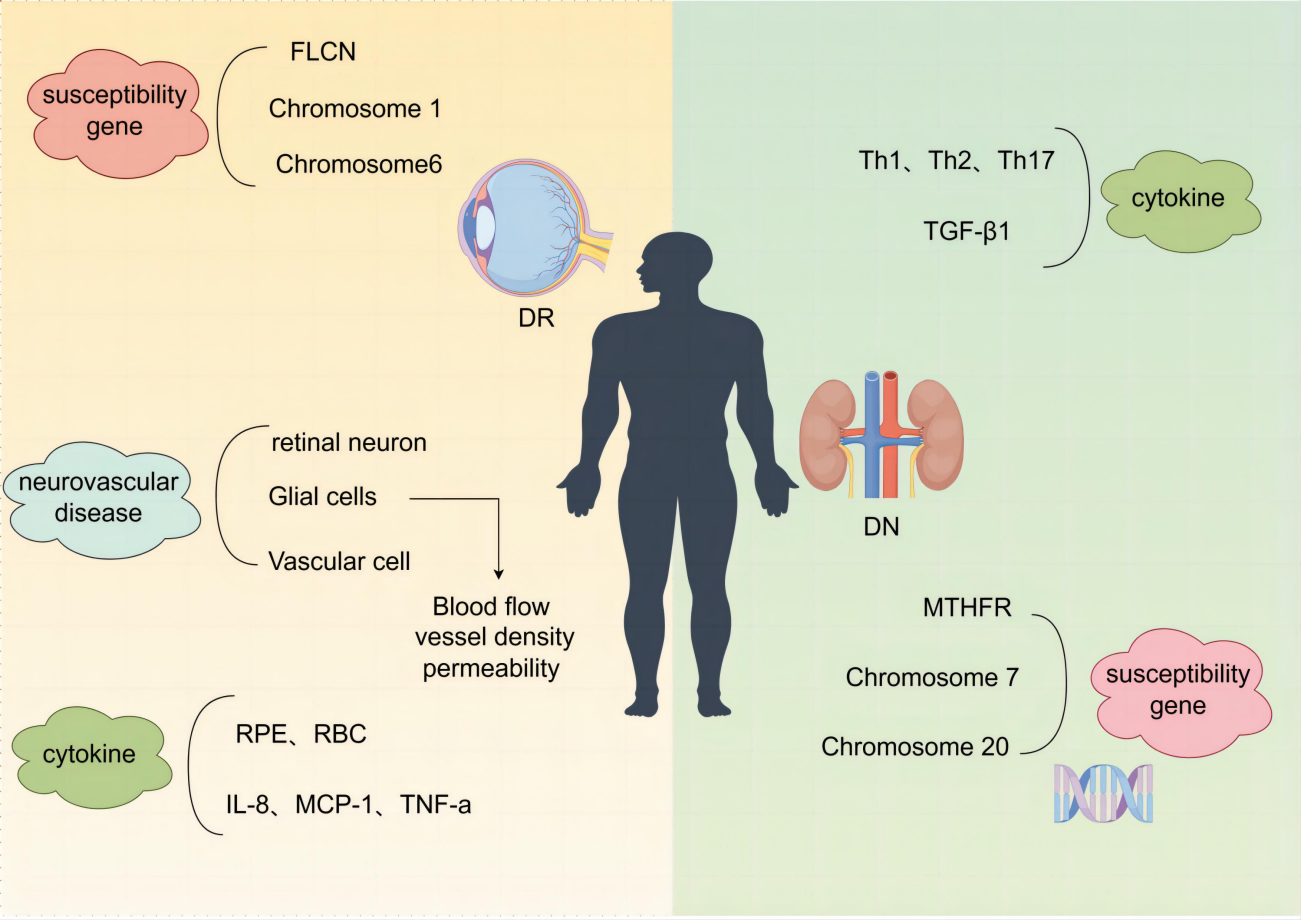
Schematic of the different mechanisms of DN and DR. DN, diabetic nephropathy; DR, diabetic retinopathy.

As two different organs, the kidney and the eye have their own unique internal environment and physiological processes, each with specific influencing molecules, and the target organs themselves have different pathological changes and responses to various influencing factors, leading to different manifestations of diabetic microangiopathic damage in the two, and at the same time, the diagnostic means of the two also make the two diseases have non-parallelism. The article describes the non-parallelism of the two diseases in terms of the differences in their causative factors, the asynchrony of the disease, the differences in mechanisms, and the different medications used (**Figure 2**).

### Asynchrony in the onset of DN and DR

#### With DN without DR

In a prospective, randomized, blinded research trial of 144 Hispanics and 671 non-Hispanic white patients with non-insulin-dependent diabetes mellitus (NIDDM), it was shown that dominant albuminuria (urinary albumin excretion rate >200 μg/min) (197) was a strong independent risk factor for DR in the Hispanic study population, whereas non-Hispanic white patients did not show such an association, and the study suggests that early care may differ between the two ethnic groups due to differences in economic level as well as healthcare, which may be a possible reason for differences in the prevalence of diabetes complications, particularly DN and DR (198). Renal biopsy is the gold standard for the diagnosis of DN, by which renal disease and its prognostic classification can be accurately diagnosed but may not be performed in diabetic patients due to the limitations of the patient’s age, comorbidities, anticoagulant therapy, and additional costs (199). By screening T2DM patients who underwent renal biopsy, 96 (38.71%) in the DN group and 152 (61.29%) in the non-diabetic renal disease (NDRD) group, it was found that DR was prevalent in 79 (82.3%) and 12 (7.9%), respectively, suggesting that diabetic patients without retinopathy were the most likely to develop NDRD, which suggests that clinically, some DN patients have no DR may be because clinically some patients diagnosed with DN did not undergo renal biopsy, and some patients with NDRD combined with IgE, for example, were included. This suggests that diabetes mellitus without retinopathy has the highest likelihood of developing NDRD, which suggests that some patients with DN are not clinically comorbid with DR possibly because renal biopsy is not practiced in some of the clinically diagnosed patients with DN, and some patients with diabetes mellitus comorbid with NDRD, e.g., IgA nephropathy, are included and therefore do not have DR (200). Of the 98 patients with simple DN, 64 (65.3%) had DR and 34 (34.7%) had no DR, and the analysis concluded that advanced age (p = 0.003) and male gender (p < 0.001) were significantly associated with DN without DR (201).

#### With DR without DN

In 138 patients with insulin-dependent diabetes mellitus aged between 25 and 34 years with onset before 30 years of age, it was found that nephropathy was rare in patients without retinopathy, but retinopathy was often seen in patients without nephropathy. In both cases, DR was often detected earlier than DN in patients with insulin-dependent diabetes mellitus (202). This may be due to the higher vulnerability of the retina compared to the kidneys and the higher detection rate of DR, as fundus photography or fundography is more accessible to perform and therefore allows for easier detection compared to renal biopsy (203). The trial studied 100 insulin-treated diabetic patients, 35% of whom had PDR without DN changes, suggesting that the two types of microangiopathy, DN and DR, may not evolve similarly (204).

### Different Pathogenesis of DN and DR

#### Different susceptibility genes

The development of DN and DR is hereditary and has familial aggregation, and the extent of disease damage is also determined by the genetic determinants of individual susceptibility as well as the presence of multiple independent risk factors (205–207). In a meta-analysis of genome-wide association studies, the folliculin gene (FLCN) was identified as a susceptibility gene for DR (208). Methylenetetrahydrofolate-reducing gene (MTHFR), a key enzyme regulating nucleotide synthesis and DNA methylation, is a susceptibility gene for DN and may be a risk factor for DN in white people and Africans (209). Chromosome 7 and chromosome 20 are susceptibility genes for DN (210). Chromosome 1 (211) and chromosome 6 (212) are susceptibility genes for DR.

#### DR: diabetic microvascular complications, neurovascular lesions

The American Diabetes Association defines DR lesion as a highly tissue-specific neurovascular complication (213, 214), a neurovascular disease resulting from the destruction of the retinal neurovascular unit (NVU) (214), which consists of retinal neurons, glial cells, and vascular cells coordinately regulating blood flow, vascular density, and permeability in response to the similarly dynamic demands of the retinal neurons, through the supply of oxygen and nutrients, the recycling of neurotransmitters, and the removal of metabolic wastes, a vascular function whose fine-tuning is essential for maintaining retinal homeostasis (215). Retinal neurodegeneration is involved in the development of microvascular abnormalities as an early event in DR (215, 216). In addition to premature neuronal death, biochemical and structural changes in neurons and glial cells contribute to neurodegeneration, and the functional abnormalities that occur in DR may be due to early changes in neural organization (217).

#### Different cytokines

The growing agreement that DN is an inflammatory process is because leukocyte infiltration occurs at all stages of kidney damage (96). The involvement of proinflammatory factors, Th1, Th2, and Th17 cytokines and TGF-β1 in the development of DN predicts susceptibility and progression of DN (218, 219). Inflammatory cytokines and oxidative stress-related pathways DR in different systems such as red blood cells (RBC) and retinal pigment epithelium (RPE) cells provide important potential biomarkers (220). Some findings support the association of PDR bleeding with IL-8, MCP-1, TNF-α, and other inflammatory cytokines in atrial fluid (221).

### Different medications for DN and DR

#### DN clinical use

Treatment of DN includes strict control of blood glucose and blood pressure, restriction of protein intake, and maintenance of water–electrolyte and acid–base balance (222). In addition to hypoglycemic effects, dipeptidyl peptidase-IV (DPP-4) inhibitors exert renoprotective effects through antioxidant and anti-inflammatory mechanisms, and anti-fibrotic effects through inhibition of TGF-β-mediated signaling (223). Inhibitors of sodium-dependent glucose transporters 2 (SGLT-2) reduce hyperglycemia while preventing glomerular hyperfiltration and slowing the development of DN (224, 225). Aldosterone is pro-inflammatory and pro-fibrotic and promotes kidney injury (226), and aldosterone can elevate arterial and glomerular pressure (227). Aldosterone receptors are present in glomerular endothelial and epithelial cells, and abnormal aldosterone infusion can lead to proteinuria and glomerulosclerosis by damaging glomerular epithelial cells (228). Clinically available aldosterone receptor antagonists are used to treat DN, and proteinuria continues to be significantly reduced during spironolactone treatment; however, it should be noted that aldosterone receptor antagonists, such as spironolactone, increase the risk of hyperkalemia (229). Currently, a number of new drug targets have recently been identified against the underlying pathophysiological mechanisms of DN that may contribute to the development of new drugs to prevent renal and vascular damage and slow down the progression of DN in patients with DN, including the development of personalized medicines based on genetic and epigenetic variations (230). New therapeutic targets such as the GSK3β signaling pathway (231), NLRP3 inflammatory vesicles (232), PI3K/Akt and JAK/STAT pathways (233), endothelin receptor (234), and DPP-4 (235) are involved in the pathological progression of DN, and there is still an urgent need to identify and validate new drug targets and candidates for better DN therapy.

#### DR clinical use

The main methods of DR treatment include strict control of blood glucose, blood pressure, and blood lipids, medication to improve microcirculation and anti-neovascularization, laser therapy, and vitrectomy (236). Lipid-lowering drug fenofibrate slows DR progression through anti-inflammatory, anti-angiogenic, and retinal neuroprotective effects (237). *VEGF inhibitors*: VEGF is a hypoxia-induced angiogenic peptide (238) that mediates BRB destruction and angiogenesis in the pathogenesis of DR (239). Clinicians reduce VEGF signaling in the retina by vitreous cavity injections of anti-VEGF biologics. In a series of disease models and clinical studies, it has been shown that multiple pharmaceutical agents that successfully target VEGF significantly improve vascular permeability and retinal edema (240). Anti-VEGF drugs improve DR severity and are the first-line treatment for diabetic macular edema with PDR (241). Current anti-VEGF agents for the treatment of DR include bevacizumab, ranibizumab, and abciximab (240, 242), which have demonstrated their safety in several trials (243). Studies have shown that ranibizumab rapidly and consistently improves vision and reduces the risk of further vision loss in patients with diabetic macular edema (244). The anti-VEGF regimen is usually 1 injection per month for 3 months and every 4–6 weeks if necessary (245). Commonly used drugs for treatment with intravitreal injections of corticosteroids include dexamethasone, triamcinolone acetonide, and flurazepam (246). Studies have shown that dexamethasone is safer, has the lowest risk of causing glaucoma and cataracts, and can be classified as the drug of choice for intraocular corticosteroid injections (247, 248). Corticosteroids target pro-inflammatory mediators in DME, including IL-6, IL-8, MCP-1, ICAM-1, and TNF-α (249), producing anti-inflammatory effects through various mechanisms, including reducing the synthesis of inflammatory mediators and adhesion proteins and lowering VEGF (250). These drugs are commonly used to treat patients with refractory and vision-threatening DR and DME because of the bracketed IOP elevation and cataract risk (251).

Future directions in DR treatment will focus on the following two areas: the development of retinal imaging techniques and the discovery of new molecules for the treatment of refractory patterns of DME (252). The continued refinement of new technologies such as AI (253) and ultra-wide-angle (UWF) retinal imaging (254) provide important support for early screening for DR. New targets such as anti-VEGF (255), anti-inflammatory (249), neuroprotection (190, 194), soluble epoxide hydrolase (sEH) (256), nano molecules (52), and photo-biomodulation (PBM) (257) provide more directions for the development of new therapeutic approaches for DR and suggest that the future trend in the management of DR may be a multi-pathway targeted therapy. Among them, high treatment burden, treatment adherence problems, and under-treatment are the challenges that DR treatment needs to face.

## Summary and outlook

DN and DR, as diabetic microvascular comorbidities, are the main factors of end-stage renal disease and blindness, respectively, which bring a heavy economic burden to society. Parallelism and non-parallelism exist in the process of disease development of the two diseases, and the parallelism of the two helps to guide the co-medication of the two diseases. DN and DR homeopathic drugs can be used as a future research direction for the treatment and prevention of the two diseases to reduce the types of medication used by patients, optimize the management, alleviate the burden of hepatic and renal metabolism, and improve the patients’ quality of life, as well as further alleviate the burden of patients and the country’s medical care. Early comprehensive intervention is of great significance. Current research suggests that the two diseases are treated with polyol pathway inhibitors, antioxidants, RAS system inhibitors, and vasoprotective agents, which have a better potential for development, and in the development of new anti-inflammatory drugs, miRNA is a new therapeutic target in seeking breakthroughs. The development of drugs targeting these common molecular pathways may provide new therapeutic approaches for both diseases.

## Author contributions

ST: Writing – original draft. XA: Writing – original draft. WS: Writing – original draft. YZ: Writing – original draft. CY: Writing – original draft. XK: Writing – original draft. YS: Writing – original draft. LJ: Writing – original draft. XZ: Writing – original draft. QG: Writing – original draft. HJ: Writing – review & editing. FL: Writing – review & editing.

## Glossary

**Table:**

DN	diabetic nephropathy
DR	diabetic retinopathy
ESRD	end-stage renal disease
T1DM	type 1 diabetes mellitus
T2DM	type 2 diabetes mellitus
BRB	blood–retina barrier
RAAS	renin–angiotensin–aldosterone system
NPDR	non-proliferative diabetic retinopathy
PDR	proliferative diabetic retinopathy
VEGF	vascular endothelial growth factor
NO	nitric oxide
ROS	reactive oxygen species
AngII	angiotensin II
OS	oxidative stress
AGEs	advanced glycosylation end products
PKC	protein kinase C
NF-κB	nuclear factor κB
TXNIP	thioredoxin-interacting protein
HIF-1α	hypoxia-inducible factor 1α
KKS	kinin–kinin system
AR	aldose reductase
ALA	alpha-lipoic acid
ACEI	angiotensin-converting enzyme inhibitor
ARB	angiotensin II receptor blocker
CaD	calcium hydroxybenzenesulfonate
FLCN	folliculin gene
RBC	red blood cells
RPE	retinal pigment epithelium
DPP-4	dipeptidyl peptidase-IV
SGLT-2	sodium-glucose cotransporter protein 2
AI	artificial intelligence
HbAlc	glycosylated hemoglobin
